# Between a Rock and a Hard Place: Balancing Embolic Stroke and Intracerebral Hemorrhage Risk in Left Atrial Appendage Occlusion

**DOI:** 10.3390/jcdd13030148

**Published:** 2026-03-23

**Authors:** Juan Felipe Daza-Ovalle, Johanna Seiden, Daniel Labovitz, Erick Daniel Martinez, Deepti Athreya, Charles Esenwa

**Affiliations:** 1 Department of Neurology, Albert Einstein College of Medicine at Montefiore Health System, 111 E 210th St, Bronx, NY 10467, USA; jseiden@montefiore.org (J.S.); dlabovit@montefiore.org (D.L.); ericmartin@montefiore.org (E.D.M.); deepti.athreya@einsteinmed.edu (D.A.); cesenwa@montefiore.org (C.E.); 2 Comprehensive Stroke Center at Montefiore Health System, 3316 Rochambeau Ave, Bronx, NY 10467, USA

**Keywords:** atrial fibrillation, intracerebral hemorrhage, left atrial appendage occlusion, percutaneous left atrial appendage occlusion, anticoagulants, ischemic stroke, cerebral amyloid angiopathy, small vessel disease, direct oral anticoagulants (DOACs), cerebral microbleeds, magnetic resonance imaging, arteriovenous malformation, subarachnoid hemorrhage, subdural hematoma

## Abstract

Patients with atrial fibrillation (AF) who are not candidates for long-term anticoagulation present a complex therapeutic dilemma due to competing risks of cardioembolic stroke and intracerebral hemorrhage (ICH). This challenge is particularly pronounced in neurologically vulnerable individuals, including those with prior ICH, cerebral amyloid angiopathy (CAA), or neuroimaging markers of cerebral small vessel disease (SVD). Left atrial appendage occlusion (LAAO) has emerged as an alternative stroke prevention strategy for patients with contraindications to anticoagulation; however, optimal patient selection and post-procedural antithrombotic management remain uncertain, largely because existing bleeding risk scores inadequately capture ICH risk. Most hemorrhagic risk scores were designed to estimate systemic bleeding and demonstrate limited ability to predict ICH, as they do not incorporate hemorrhage etiology or neuroimaging features. Importantly, ICH recurrence risk varies substantially by subtype, with the highest risk observed in CAA-related hemorrhage, the lowest in hypertensive SVD, and intermediate risk in mixed or secondary etiologies. These distinctions have direct implications for anticoagulation decisions and consideration of LAAO. Finally, we synthesize contemporary evidence on ICH risk stratification, neuroimaging biomarkers, and antithrombotic strategies following LAAO. We propose a multidisciplinary, evidence-based decision-making framework integrating clinical risk scores, neuroimaging findings, and hemorrhage phenotype to support individualized stroke prevention strategies in high-risk patients with AF.

## 1. Introduction

Management of atrial-fibrillation-associated stroke in patients for whom anticoagulation is contraindicated remains a complex therapeutic dilemma. These patients and their providers face a fundamental clinical challenge characterized by high risk of recurrent cardioembolic stroke on one hand and an increased risk of intracerebral hemorrhage (ICH) on the other. This tension represents a classic “rock-and-hard-place” scenario in which strategies to mitigate embolic stroke risk simultaneously increase hemorrhage risk. This paper synthesizes the current evidence and introduces a multidisciplinary decision-making algorithm to guide the use of left atrial appendage occlusion (LAAO) in neurologically vulnerable individuals.

A major limitation in current decision-making is the absence of validated tools specifically designed to predict ICH. Common bleeding risk scores, including HAS-BLED, HEMORR2HAGES, and ATRIA, were developed to estimate systemic bleeding and do not incorporate neuroimaging biomarkers or established neurologic risk factors such as small vessel disease SVD or cerebral amyloid angiopathy CAA [1]. Although HAS-BLED demonstrates modest predictive ability for ICH, its discriminative performance remains limited [2], and other scores have not consistently demonstrated independent predictive values for ICH [2,3]. These gaps underscore the need for a more phenotype-driven and multidisciplinary approach to stroke prevention in patients at elevated hemorrhagic risk.

In this review, we synthesize contemporary evidence on ICH risk stratification, hemorrhage phenotyping, and LAAO outcomes, and we propose a structured, multidisciplinary framework to support individualized decision-making in this high-risk population.

## 2. Scope and Methodology of the Review

This manuscript represents a narrative, expert-driven review rather than a formal systematic review. The relevant literature was identified through focused searches of main databases such as PubMed, Scopus, and Embase, covering publications up to March 2026. The search emphasized randomized controlled trials, large observational studies, meta-analyses, and contemporary professional society guidelines addressing AF, ICH, CAA, hemorrhagic risk stratification, and LAAO. Articles were selected based on clinical relevance, methodological rigor, and applicability to neurologically vulnerable populations. No predefined systematic protocol, formal inclusion or exclusion criteria, or PRISMA-based screening process was applied.

## 3. Intracerebral Hemorrhage Risk Stratification and Classification

ICH risk varies substantially across the five major etiologic subtypes between arteriolosclerosis, CAA, mixed SVD, other rare forms of SVD, and secondary causes such as macrovascular lesions or tumors [4].

Hypertensive SVD is associated with a low annual risk of ICH recurrence, estimated at 0.6–1.1 percent per year, with recurrences typically involving deep brain structures such as the basal ganglia, thalamus, or brainstem [5,6,7].

CAA confers the highest risk of recurrent ICH, with annual recurrence rates of approximately 7.4–8.5 percent, rising to 15–20 percent per year in patients with multifocal cortical superficial siderosis (cSS) and multiple lobar cerebral microbleeds (CMBs) [5,8]. Conversely, mixed SVD is associated with an intermediate recurrence risk, estimated at approximately 1.8 percent per year, reflecting the coexistence of both deep and lobar ICH on neuroimaging [6,9].

Other rare forms of SVD, including genetic or inflammatory etiologies, demonstrate variable and less well-defined recurrence risk, which is generally considered low to intermediate in the absence of specific high-risk features. Secondary causes of ICH, such as macrovascular lesions or tumors, are associated with a low risk of recurrence following definitive treatment, whereas untreated lesions persistently carry elevated risk [5,6].

### 3.1. Cardioembolic Stroke Risk in Atrial Fibrillation (AF)

Cardioembolic stroke accounts for 20–25% of all ischemic strokes and is associated with worse clinical outcomes compared to other ischemic stroke subtypes [10,11]. AF is the most common cause of cardioembolic stroke and is responsible for 15% of all strokes worldwide [11]. Given the high morbidity and mortality from AF-related stroke, accurate assessment of thromboembolic risk is central to long-term management.

The CHA_2_DS_2_-VASc score is a clinical prognosticator for 1-year risk of thromboembolic events in untreated patients with non-valvular AF [12]. In this context, a sex-neutral CHA_2_DS_2_-VASc score was used, reflecting ongoing uncertainty regarding female sex as an independent stroke risk factor in AF [13,14,15,16]. Based on the CHA_2_DS_2_-VASc risk stratification system, all patients with AF and previous history of ischemic stroke or transient ischemic attack (TIA) should be recommended for lifelong oral anticoagulation therapy with a direct oral anticoagulation DOAC. Conversely, antiplatelets are not recommended for secondary stroke prevention in the setting of AF [17].

### 3.2. LAAO as a Non-Pharmacologic Strategy

In non-valvular AF, over 90% of thromboemboli originate in the left atrial appendage (LAA) [18,19,20,21]. The first percutaneous left atrial appendage occlusion (LAAO) system, WATCHMAN, was approved in 2015 by the U.S. Food and Drug Administration as an alternative to long-term oral anticoagulation therapy in selected patients. Additional occlusion devices, such as Amulet (Abbott), have since been developed and evaluated in randomized controlled trials [16]. Other non-percutaneous surgical options are available and capable of LAAO, ligation or excision; however, they are not FDA-approved for stroke prevention.

An important consideration when recommending LAAO is the risk of device-related thrombosis (DRT). DRT is highest before full endothelialization, which may take several weeks to months [17,22,23,24]. Early post-LAAO protocols recommended 45 days of warfarin therapy (INR target 2–3) or a DOAC plus aspirin (ASA), followed by transesophageal echocardiography to confirm device seal, dual antiplatelet therapy (DAPT) for up to 6 months, and lifelong ASA thereafter [17,22,23,25,26,27]. Since initial development, alternative regimens with DOAC alone or DAPT have been adopted with comparable efficacy and reduced bleeding in select high-risk patients [25,27,28,29].

### 3.3. High ICH Risk After LAAO

Despite the expanding use of LAAO in high-risk patients, a subset exists in whom the risk of major bleeding is considered so extreme that even a short course of anticoagulation is contraindicated. Examples include patients with severe gastrointestinal bleeding risk, high HAS-BLED scores, or substantial cerebral microhemorrhage burden [3,30]. Assessing ICH risk following LAAO is therefore essential, yet clinical guidelines defining high ICH risk in the context of LAAO planning are lacking. Additionally, the optimal post-LAAO antithrombotic strategy for patients with absolute contraindications to anticoagulation remain uncertain.

**Figure 1:**
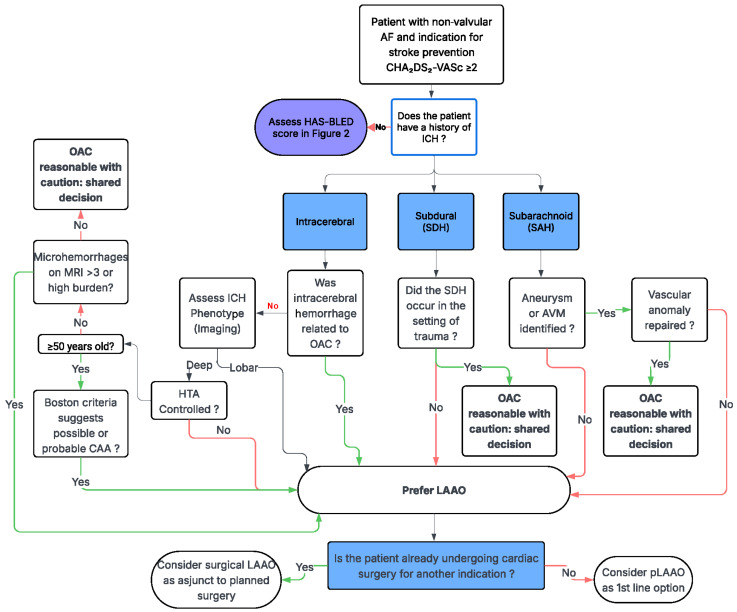
Phenotype-guided framework for stroke prevention in atrial fibrillation with prior intracranial hemorrhage. This figure presents a conceptual, phenotype-driven framework for patients with non-valvular atrial fibrillation (AF) and prior intracranial hemorrhage (ICH), distinguishing intracerebral, subdural (SDH), and subarachnoid (SAH) hemorrhages and incorporating anatomic location, underlying etiology, blood pressure control, Boston criteria for cerebral amyloid angiopathy (CAA), and cerebral microbleed burden on magnetic resonance imaging (MRI) to estimate recurrence risk. Deep hypertensive ICH with controlled risk factors may permit cautious consideration of oral anticoagulation (OAC), whereas lobar ICH, probable CAA, or high microbleed burden may favor consideration of left atrial appendage occlusion (LAAO); SDH and SAH management depends on traumatic versus spontaneous origin and vascular repair status. This algorithm has not undergone prospective or retrospective validation and should be considered hypothesis-generating; it is intended to facilitate multidisciplinary evaluation and shared decision-making rather than replace guideline-directed, individualized clinical management.

**Figure 2:**
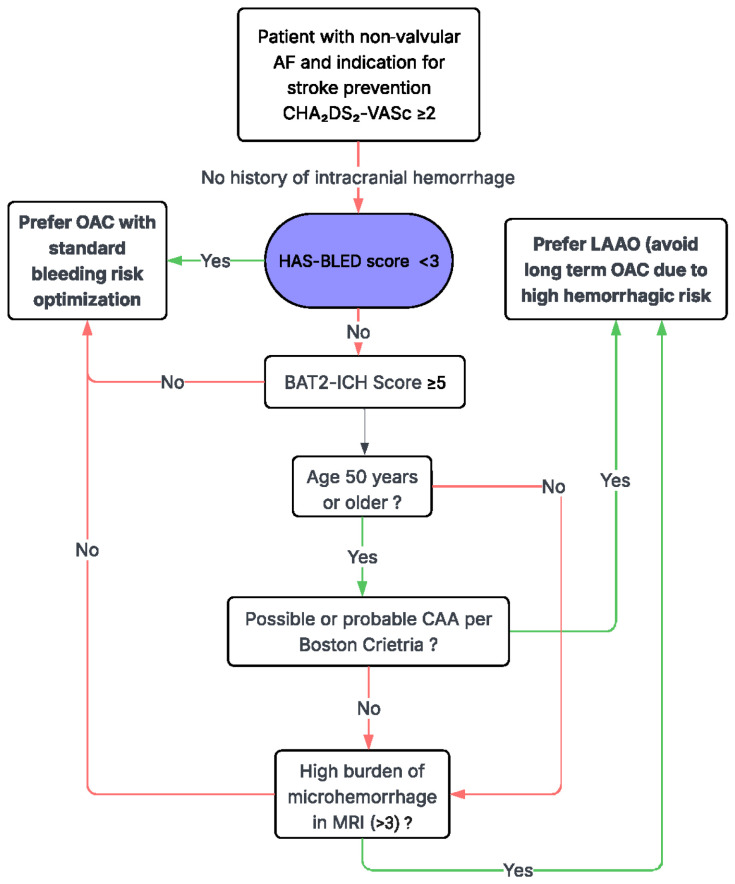
Conceptual decision-support framework for stroke prevention in atrial fibrillation without prior intracranial hemorrhage. This figure presents a conceptual, phenotype-informed framework for stroke prevention in patients with non-valvular atrial fibrillation (AF) without prior intracranial hemorrhage (ICH), integrating clinical bleeding risk scores (e.g., HAS-BLED) with neuroimaging markers such as cerebral microbleeds and features suggestive of cerebral amyloid angiopathy (CAA) to refine assessment of intracerebral hemorrhage risk. Patients with lower neurologic vulnerability may be managed with oral anticoagulation (OAC) and optimization of modifiable bleeding risk factors, whereas those with higher cerebral bleeding susceptibility may be considered for left atrial appendage occlusion (LAAO) to avoid long-term anticoagulation exposure. This algorithm has not undergone prospective or retrospective validation and should be regarded as hypothesis-generating; it is intended to support multidisciplinary clinical discussion rather than to function as a guideline-level recommendation or replace individualized, guideline-directed management.

Herein, we present a summary of the available evidence related to ICH risk stratification and emerging data supporting shorter DAPT duration or minimal anticoagulation strategies for high-risk patients [22,23,24,28]. We subsequently propose an evidence-based multidisciplinary algorithm (Figure 1 and Figure 2) to guide decision-making and evaluate eligibility for LAA closure in neurologically vulnerable patients.

#### Pathophysiology of Intracerebral Hemorrhage

ICH results from bleeding directly into the brain parenchyma following rupture of a structurally compromised cerebral blood vessel. Despite the lack of agreement regarding etiologic classification systems [31], spontaneous ICH is broadly classified into primary ICH, resulting from the rupture of small vessels from SVD, or secondary ICH, resulting from identifiable structural, traumatic, neoplastic, hematologic, or other rare underlying processes [4,31,32].

Hypertensive cerebral vasculopathy remains the leading cause of primary ICH [4]. Deep ICH associated with hypertensive arteriopathy predominantly affects the basal ganglia, thalamus, brainstem, or deep cerebellum and exhibits arteriolar lipohyalinosis, fibrinoid necrosis, and occasional Charcot–Bouchard microaneurysms rather than amyloid deposition [33,34]. Gross pathology and imaging typically reveal well-circumscribed hematomas centered in deep brain structures, frequently with intraventricular extension [33,34,35,36].

The pathophysiology involves long-standing disorganization and degeneration of the vessel walls of small penetrating arteries and arterioles due to lipohyalinosis, leading to deep-brain vessel rupture [33]. CAA, diagnosed using the Boston Criteria 2.0 [37], is another important etiology of primary ICH, particularly lobar hemorrhages in older adults. It is characterized by progressive vessel wall weakening and subsequent increased rupture risk due to β-amyloid accumulation in leptomeningeal and cortical arterioles and capillaries [38,39].

Pathological examination demonstrates smooth muscle cell loss, vessel wall thickening, and frequent cSS, as observed in operative, biopsy, and autopsy specimens [33,35,36,40]. Gross pathological and surgical images often show irregularly bordered lobar hematomas, sometimes with subarachnoid extension, reflecting diffuse cortical vessel fragility [33,34]. In patients over 60 years, the presence of apolipoprotein (Apo) E2 and ApoE4 alleles has been strongly associated with increased vascular β-amyloid accumulation and a greater hemorrhagic risk [33].

Together, these findings highlight distinct gross, histopathological, and anatomic features that differentiate CAA-related lobar hemorrhage from hypertensive deep ICH [33,34,35,36,40].

Based on limited evidence from small case series, the presence of an unruptured arteriovenous malformation (AVM) does not constitute an absolute contraindication to anticoagulation [41]. Nevertheless, certain AVMs may carry a higher rupture risk due to features such as deep location or deep venous drainage leading to local venous hypertension, that promote vessel wall remodeling and fragility [42,43,44]. Hemorrhage occurring while on anticoagulation may further increases hematoma size and damage [43,45,46,47]. In addition, vascular lesions such as AVMs and cavernous malformations, constitute important etiologies of secondary ICH, as summarized in the CLAS-ICH classification system’s listing of macrovascular etiologies [4].

## 4. Secondary Risk of ICH

A pooled analysis reported an annual recurrence rate of 7.4% for CAA-related ICH, compared with 1.1% per year for ICH not attributed to CAA [48]. Consistent with this distinction, lobar ICH carries a substantially higher risk of recurrence than deep ICH [49]. This difference is also reflected in an analysis published in 2021 which reported an absolute recurrence rate of 5.1 per 100 patient-years after lobar ICH, compared with 1.8 per 100 patient-years after deep ICH, corresponding to a more than threefold increased risk of recurrence associated with lobar ICH (hazard ratio 3.2; *p* = 0.001) [50].

General contraindications to long-term anticoagulation include thrombocytopenia, severe hepatic impairment, advanced chronic kidney disease, active major bleeding, and high risk or history of recent or recurrent ICH [51,52,53,54], among others.

### 4.1. Hemorrhagic Risk Stratification: Limitations of Traditional Bleeding Scores and Emerging ICH-Specific Tools

To inform anticoagulation decisions in patients with AF, several clinical bleeding risk scores including HAS-BLED, HEMORR2HAGES, ATRIA, and ORBIT are commonly applied [55,56,57,58,59,60]. Each was validated using data from clinical cohorts or registries of patients with AF, specifically to provide a practical tool for quantifying bleeding risk in the context of anticoagulation therapy. These tools were developed to estimate major systemic bleeding risk and share overlapping variables with thromboembolic risk scores, including age, hypertension, and prior stroke, underscoring that patients at elevated bleeding risk often simultaneously face substantial embolic risk [61,62]. In clinical practice, this overlap frequently prompts consideration of non-pharmacologic stroke prevention strategies, particularly in patients with prior ICH or other markers of neurologic vulnerability [22].

However, these scores were not specifically designed to predict ICH and do not incorporate neuroimaging biomarkers such as CMBs, cSS, or markers of CAA. Among available tools, HAS-BLED is the only score to demonstrate statistically significant predictive performance for ICH in anticoagulated AF populations. In the AMADEUS study (a randomized, multicenter clinical trial), the HAS-BLED score achieved a c-statistic of approximately 0.75 (95% CI 0.56–0.95) for ICH prediction and was the only evaluated score to show significant discrimination for this outcome [2]. Nevertheless, its overall performance across broader and higher-risk populations has been more modest. In a cohort of patients after first spontaneous ICH, the HAS-BLED score demonstrated a c-statistic of 0.54 (95% CI 0.50–0.59) for recurrent hemorrhage prediction [63]. Similarly, in a large Danish nationwide cohort, recalibration of HAS-BLED modestly improved prediction of major bleeding to a c-statistic of 0.616 (95% CI 0.610–0.622) [64]. Pooled and multicenter analyses report c-indices ranging from approximately 0.41 to 0.66 for intracranial or major bleeding events, with most values remaining below 0.70, reflecting limited discriminatory capacity [2,3,65].

Importantly, ICH carries substantially higher one-year mortality compared with major systemic hemorrhage [66,67], highlighting the need for more precise risk stratification strategies. Emerging tools such as the updated Boston Criteria Version 2.0 incorporate hemorrhagic and MRI-based markers to improve identification of CAA and enhance ICH discrimination, with reported c-indices around 0.75 (95% CI 0.67–0.80) [68]. These approaches are best viewed as complementary to traditional bleeding scores, introducing phenotype-driven and imaging-informed assessment into clinical decision-making.

### 4.2. Emerging ICH-Specific Risk Stratification Tools

The BAT2 score combines clinical factors, including age, underweight status, hypertension, and type of antithrombotic therapy, and imaging, with neuroimaging markers such as CMBs, lacunes, and deep white matter hyperintensities identified on MRI to estimate ICH risk. It stratifies risk in three practical categories. Patients with a score 0–1 are low risk (annual ICH risk 0.1–0.2% [69]; scores 2 to 3 are in the intermediate-risk range (annual ICH risk 0.5–0.7% per year [69]); scores equal to or above 4 are high risk (annual ICH rate up to 2%) [69].

Although an annual ICH risk of approximately 2% alone may not be sufficient to justify LAAO, it is important to clarify that the BAT2-ICH score does not differentiate between lobar and deep CMBs when estimating hemorrhagic risk [69]. While the score incorporates the presence and burden of CMBs as a predictor, it relies on total microbleed count rather than anatomical distribution [69]. This limitation is clinically relevant, given the markedly different recurrence risks associated with lobar microbleeds related to CAA compared with deep microbleeds associated with hypertensive arteriopathy.

While anticoagulation may be used in low- and medium-risk groups, in the high-risk group, clinicians may consider surgical ligation or excision of the LAA, which typically does not require routine anticoagulation or DAPT unless there are other compelling indications [16,22]. However, the procedural risk profile of surgical ligation/excision of LAA differs from the percutaneous LAAO approach and should be carefully weighed in the decision-making process, depending on the other comorbidities. This approach allows care to be tailored to each patient’s bleeding and stroke risk in a more detailed and individualized way.

Finally, the SoSTART collaboration demonstrated in a non-inferiority trial that patients who restarted anticoagulation after ICH had a higher recurrence rate of ICH (8%) compared to patients who did not resume anticoagulation (4%) [70]. These findings failed to demonstrate non-inferiority of restarting anticoagulation within the first two years following an ICH [33,70]. Although the randomized SoSTART trial did not demonstrate a statistically significant reduction in ischemic events, the study also did not differentiate between deep and lobar ICH subtypes, which are important predictors of recurrent hemorrhagic risk [70].

#### 4.2.1. History of Subdural Hematoma (SDH)

The cumulative risk of recurrent SDH is estimated at approximately 9% within 4 weeks of the initial bleeding event, increasing and stabilizing at around 14% after one year [71]. In the setting of oral antithrombotic therapy (either antiplatelet or anticoagulant), a meta-analysis from 2019 showed a significantly higher risk of SDH recurrence in patients on anticoagulation (OR 1.41, 95% CI 1.10–1.81; *p* = 0.006) as well as antiplatelet therapy (OR 1.23, 95% CI 1.01–1.49; *p* = 0.03) [72]. Overall survival was not adversely affected by antithrombotic therapy, despite its association with recurrence. Specifically, the study demonstrated that both anticoagulants and antiplatelet agents similarly increased the likelihood of chronic SDH recurrence; however, neither class was associated with a significant increase in mortality, indicating no measurable impact on overall survival. This finding suggests that while antithrombotic use may predispose patients to reoperation, it does not translate into worse survival outcomes [72].

#### 4.2.2. History of Subarachnoid Hemorrhage (SAH)

It is thought that oral anticoagulation in patients with a prior SAH carries a low but clinically meaningful risk of recurrent bleeding. However, available data regarding isolated SAH remain limited and are largely extrapolated from broader studies about ICH [73,74]. In the COCROACH study, subgroup evaluations were performed based on age (cut-off 75 years), sex, interval since ICH presentation, thromboembolic risk as measured by the CHA_2_DS_2_-VASc score (≤4 vs. >4), and hemorrhage subtype, including non-aneurysmal SAH [73]. No significant interaction between treatment and any subgroup was identified for the primary outcome, suggesting comparable efficacy and safety of anticoagulation across these groups. Collectively, current data indicate that the overall clinical benefit of anticoagulation after SAH in patients with AF does not vary significantly by age, comorbidity burden, or hemorrhage characteristics [73].

#### 4.2.3. Controlled vs. Non-Controlled Hypertension

Uncontrolled hypertension remains the strongest modifiable risk factor for both initial and recurrent ICH in all studied populations [33]. In one study, the event rate for lobar ICH was 84 per 1000 person-years for patients with inadequate blood pressure control, compared with 49 per 1000 person-years among those with adequate BP control. For deep ICH, the event rate was 52 per 1000 person-years with inadequate BP control and 27 per 1000 person-years with adequate BP control [75]. Although Biffi et al. did not explicitly report numeric thresholds for adequate versus inadequate blood pressure control, classification was based on contemporaneous American Heart Association and American Stroke Association recommendations during follow-up, which, at that time, set goal systolic blood pressure below 140 mm Hg and diastolic blood pressure below 90 mm Hg [76].

## 5. Surgical Ligation or Excision vs. Percutaneous LAA Occlusion (pLAAO)

LAA closure can be performed surgically through excision, internal ligation, or stapling or performed percutaneously with a closure device. While both surgical and percutaneous options are supported by the current guidelines [16], the choice between surgical LAAO and pLAAO depends on whether the patient already needs open cardiac surgery for other conditions such as valvular replacement.

### 5.1. Percutaneous LAA Occlusion Versus Oral Anticoagulation

The PROTECT AF trial was the first randomized study comparing pLAAO using the Watchman device with warfarin in patients with non-valvular AF [77]. The trial met its prespecified criterion for non-inferiority for the primary composite endpoint of stroke, systemic embolism, and cardiovascular death. However, careful interpretation of the safety profile is warranted.

In the initial report, primary safety events occurred more frequently in the device arm (rate ratio 1.69), largely driven by peri-procedural complications, including serious pericardial effusion, procedure-related stroke, and device-related events [77]. Notably, many adverse events in the device group were clustered around the time of implantation, reflecting a front-loaded procedural hazard, whereas warfarin-associated complications accumulated more gradually over time [26,77]. These findings underscore that pLAAO involves an early procedural risk that must be weighed against the long-term risks of chronic anticoagulation.

The enrolled population also included a substantial proportion of patients at relatively lower thromboembolic risk by contemporary standards, with approximately one-third of patients in the device arm (33.9%) and over one-quarter in the warfarin arm (27.0%) having a CHADS_2_ score of 1 [77]. This distribution may limit direct extrapolation to higher-risk populations commonly encountered in current practice, particularly patients with prior ICH or suspected CAA.

After an extended follow-up with a mean of 3.8 years, the authors endorse that pLAAO met criteria for non-inferiority and statistical superiority for the composite endpoint (2.3 vs. 3.8 events per 100 patient-years; relative risk 0.60) [26]. However, the observed long-term advantage was driven predominantly by reductions in hemorrhagic stroke and cardiovascular mortality, while ischemic stroke rates were not reduced and were numerically higher in the device arm [26]. Therefore, the term “superiority” applies specifically to the composite endpoint and should not be interpreted as unequivocal superiority in ischemic stroke prevention.

It is also noteworthy that the rate of ICH in the warfarin arm of PROTECT AF was higher than typically observed in some contemporary anticoagulation trials [26]. This may have influenced the magnitude of the relative benefit observed during long-term follow-up and further supports a cautious interpretation of superiority claims. Taken together, the PROTECT AF data suggest a trade-off: pLAAO carries a higher early procedural risk but may reduce long-term hemorrhagic stroke and cardiovascular mortality compared with warfarin [26,77]. The long-term composite benefit should be understood within the context of procedural risk, patient selection, and comparator performance. Accordingly, while pLAAO represents a potential alternative to chronic warfarin therapy in selected patients, its interpretation requires balanced appraisal rather than extrapolation beyond the populations studied.

Following PROTECT AF77, the PREVAIL trial was designed as a confirmatory randomized controlled study to further evaluate the safety and efficacy of pLAAO compared with warfarin [78]. Although procedural safety improved with increasing operator experience, PREVAIL did not achieve non-inferiority for one of its co-primary efficacy endpoints, particularly the early ischemic event endpoint [78]. This finding tempers the strength of confirmatory randomized evidence and highlights the endpoint-specific nature of trial interpretation. Moreover, both PROTECT AF and PREVAIL used warfarin as the comparator [77,78]. Given that DOACs have since become standard therapy and are associated with lower rates of ICH, non-inferiority relative to warfarin does not necessarily imply equivalence to contemporary anticoagulation strategies. Overall, these data support a cautious and nuanced interpretation of the randomized evidence base for LAAO.

#### Observational Evidence: LAAO Versus Oral Anticoagulation

The STR-OAC LAAO study was an international, propensity score–matched analysis comparing patients undergoing LAAO after a thromboembolic event despite oral anticoagulation with patients undergoing LAAO for a formal contraindication to oral anticoagulation (EWOLUTION registry) [79,80]. Importantly, only technically successful implantations were included, and procedural failures were excluded from outcome analyses. After matching (438 pairs), ischemic stroke rates were not significantly different between cohorts (2.5% vs. 1.9%; HR 1.37, 95% CI 0.72–2.61) [80]. Although propensity matching equalized overall thromboembolic risk scores, prior ischemic stroke remained more prevalent in the STR-OAC cohort, reflecting intrinsic differences in clinical presentation. The STR-OAC cohort also demonstrated a higher composite thromboembolic risk and a lower major bleeding rate, consistent with the distinct baseline risk profiles of the two populations [80].

All-cause mortality was high in both groups (4.3% vs. 6.9%), with higher mortality observed in the EWOLUTION registry cohort [80]. As discussed by the authors, this difference may relate to variations in baseline comorbidity and implantation period rather than treatment effect. Notably, the matched STR-OAC analysis did not include a contemporaneous oral anticoagulation-only comparator for ischemic stroke; instead, relative risk reductions were calculated using risk score-based historically expected event rates rather than direct randomized comparison [80]. Therefore, while observational data suggest that LAAO after anticoagulation failure may provide ischemic protection comparable to that seen in guideline-indicated populations, these findings should be interpreted cautiously given selection bias, such as successful procedures, residual confounding despite matching, and the absence of randomized evidence.

### 5.2. Patient Selection for Percutaneous Versus Surgical LAA Occlusion

While direct comparison between pLAAO and surgical LAAO is not available, pLAAO is thought to be best suited for patients with AF and elevated CHA_2_DS_2_-VASc scores who do not require open cardiac surgery, as well as patients with non-valvular and sustained AF with a contraindication to oral anticoagulation [16,81]. Recently, the addition of surgical atrial ligation was shown to be beneficial in patients already on anticoagulation in the STR-OAC pLAAO and LAAOS III trials.

### 5.3. LAA Occlusion in Patients Receiving Anticoagulation

While pLAAO is primarily indicated for patients with a true contraindication to long-term anticoagulation in the context of stroke prevention and non-valvular AF, the STR-OAC pLAAO cohort study published in 2024 provided further insight into the extra benefits in patients already on anticoagulation. In this study of 433 anticoagulated patients who underwent pLAAO, the incidence of ischemic stroke was 2.8% per patient-year, compared with 8.9% per patient-year in the anticoagulation-only control group [79]. The anticoagulation-only group demonstrated a shorter time to first ischemic stroke than the pLAAO group, with a significant difference between groups (hazard ratio of 0.33; 95%, confidence interval of 0.19–0.58; *p* < 0.001) [79]. These findings suggest that for patients with non-valvular AF who experience thromboembolic events despite oral anticoagulation therapy, pLAAO is associated with a substantially reduced risk of ischemic stroke compared with continued anticoagulation alone [79].

The LAAOS III was a multicenter, randomized clinical trial that included 2379 participants with AF and a CHA2DS2-VASc score of at least 2. This study showed that surgical LAAO provides an additive reduction in the risk of stroke and systemic embolism by 33% (hazard ratio 0.67, 95% CI 0.53–0.85), with no increase in perioperative complications, major bleeding, or heart failure [82,83,84]. LAAOS IV (NCT05963698) is an ongoing study evaluating whether pLAAO using the WATCHMAN FLX device can further reduce the risk of ischemic stroke or systemic embolism in patients with AF who remain at high risk despite ongoing oral anticoagulation therapy.

## 6. Devices for Percutaneous LAA Occlusion (pLAAO)

Watchman and Amulet are the only FDA-approved devices for pLAAO in the United States [16,85] for patients with non-valvular AF and contraindication for long-term oral anticoagulation [16]. These devices are endorsed in the joint clinical practice guidelines for AF management issued by the American College of Cardiology, the American Heart Association, the American College of Chest Physicians, and the Heart Rhythm Society [16]. The choice of device depends on individual patient anatomy, operator expertise, and institutional resources [86]. After device placement, epithelialization plays a vital role in the success of pLAAO devices, as it converts the device surface from a thrombogenic foreign material into a biologically inert, endothelial-covered structure, thereby minimizing the risk of DRT formation and subsequent late embolic complications [87,88].

For the Watchman device, post-implant therapy protocol calls for 45 days on warfarin and ASA, followed by DAPT for six months, and then ASA monotherapy [85]. However, recent evidence indicates that using a DOAC or warfarin alone after the placement of the WATCHMAN FLX has been shown to lead to lower bleeding risk and fewer thrombotic events compared to DOAC and ASA [89]. Additionally, DAPT for 45 days is also an option for patients unable to tolerate anticoagulants [22]. Similarly, the Amulet device requires DAPT for 45 days to 6 months, followed by ASA monotherapy [90]. This regimen aligns with guideline recommendations and real-world data, demonstrating similar rates of DRT to the widely used Watchman device [85,90]. A concise summary of commonly used post-LAAO antithrombotic regimens, including standard protocols and alternative strategies for patients at high bleeding risk, is provided in Table 1.

### Percutaneous LAA Occlusion vs. Surgical LAA Occlusion

When comparing pLAAO with non-percutaneous surgical LAAO, the advantages of the percutaneous approach beyond a reduction in recurrent ischemic stroke include its minimally invasive nature and faster recovery, as it avoids open-heart surgery and cardiopulmonary bypass. Furthermore, pLAAO can be offered to patients who do not otherwise require cardiac surgery, thereby broadening access to stroke prevention, whereas surgical LAAO is typically limited to patients already undergoing cardiac operations.

In addition, the percutaneous approach is associated with lower perioperative morbidity, including reduced rates of infection, bleeding, and arrhythmia compared with major cardiac surgery, and is feasible in patients who are poor surgical candidates because of comorbidities or frailty [106]. Although pLAAO has demonstrated non-inferiority to surgical LAAO and to oral anticoagulation for the prevention of stroke and systemic embolism in randomized and observational studies, whether the results of LAAOS III are applicable to pLAAO remains unclear [106].

Despite these advantages, percutaneous approaches have been linked to a high rate of procedural failures [107,108] in addition to other risks such as cardiac perforation [96,109]. The most frequently reported complications associated with pLAAO include pericardial effusion, occurring in approximately 2.5–2.7% of cases, followed by vascular access site complications in about 2.5% [79].

DRT represents an important peri-procedural risk, with an estimated annual incidence of 7.2% [110]. In addition to DRT, other peri-procedural risks include ischemic stroke, TIA, major bleeding, and death occurring during or shortly after pLAAO, typically within the first 7–30 days [104,110]. Although these major adverse events are confined to a brief procedural window, an estimated annualized risk is often reported in studies to facilitate comparison with long term event rates associated with chronic therapies such as oral anticoagulation and to contextualize procedural risk within a yearly framework familiar to clinicians. Other additional peri-procedural risks include peri-device leak, which may reduce procedural efficacy, as well as infection and arrhythmias, although these events are comparatively less frequent [24].

## 7. Non-Percutaneous Options for LAAO

Surgical occlusion of the LAA can be performed through epicardial and endocardial approaches, with or without the aid of specialized devices [111]. However, excision remains the most effective and definitive surgical method, as suture-based occlusions (epicardial or endocardial) are often associated with a high incidence of persistent, residual, or recurrent connections between the LAA and the left atrium [111].

The AtriClip is a non-percutaneous clip that is placed pericardially at the base of the LAA during an invasive surgery or thoracoscopy and is associated with high rates of complete occlusion [84]. Nevertheless, complications such as pericardial effusion can occur after AtriClip placement and may be difficult to predict [112]. Other common complications are the development of tachyarrhythmias and respiratory dysfunction in patients with borderline forced expiratory volume in 1 s (FEV1) [112].

Systematic reviews indicate that most patients discontinue anticoagulation following AtriClip placement, with a very low incidence of stroke or TIA (0.2–1.5 per 100 patient-years) [97]. While current evidence suggests that ongoing anticoagulation may not be necessary after epicardial clipping, standardized guidelines regarding postoperative anticoagulation management in these patients still recommend it [96]. There are no trials comparing the AtriClip with the WATCHMAN or Amulet devices.

## 8. LARIAT as a Hybrid Epicardial–Endocardial Approach for LAAO

The LARIAT suture delivery device (SentreHEART, Inc., Redwood City, CA, USA) is a non-implant, suture-based system that achieves LAA exclusion through a combined endocardial and epicardial approach [113]. Long-term data from a multicenter real-world cohort with a mean follow-up of 6.5 years demonstrated complete closure in 93% of cases and significantly lower rates of thromboembolic events (1.9% vs. 24%), bleeding (9.2% vs. 24.4%), and mortality (5.6% vs. 20%) compared with screened but anatomically ineligible controls [114]. The overall procedural complication rate was 8.3%, including cardiac perforation and pericardial complications [114,115].

However, careful interpretation is warranted. In this cohort, CHADS2 scores <2 were present in 22.2% of patients in the LARIAT group and 28.9% of controls [114], indicating that a substantial proportion of patients were at intermediate rather than exclusively high thromboembolic risk. Baseline warfarin exposure was common, reported in 78.7% of patients in the LARIAT group and 82.2% in controls [114], and in the initial prospective experience, 94% of patients were receiving warfarin at enrollment [94]. These findings underscore that many patients were not absolute contraindication cases but rather individuals with anticoagulation-related challenges.

Post procedural antithrombotic therapy was not standardized. Most patients discontinued long-term anticoagulation and were managed with single antiplatelet therapy [114]. In a prospective cohort including 428.4 patient-years of follow up, the observed thromboembolism rate was 0.6%, corresponding to an estimated 81% reduction compared with expected risk, while severe bleeding occurred in 0.8%, reflecting an estimated 78% reduction, and overall mortality was 1.6% [116]. These observational findings support the feasibility of anticoagulation discontinuation after successful LARIAT closure in selected patients.

Nevertheless, the current evidence base for LARIAT remains derived from nonrandomized cohorts. Although further randomized evaluation such as the aMAZE trial (randomized LARIAT adjunctive study; incomplete outcome data) was anticipated [114,117], no completed randomized controlled trial has directly compared LARIAT-based ligation with contemporary oral anticoagulation for stroke prevention. Therefore, potential selection bias, anatomical exclusions, and heterogeneity in post-procedural management must be considered, and important clinical uncertainties persist regarding comparative efficacy, patient selection, and optimal long-term antithrombotic strategy.

## 9. Discussion

ICH risk is shaped by hemorrhage etiology, anatomic location, neuroimaging markers of SVD, and the clinical context in which prior bleeding occurred. The decision-making algorithms presented integrate these factors into a structured, stepwise framework that bridges neurologic and cardiovascular risk assessment. Beginning with standard thromboembolic and bleeding risk scores, the algorithm then incorporates hemorrhage subtype (intracerebral, subdural, or subarachnoid), hemorrhage location (lobar versus deep), anticoagulation association, and advanced neuroimaging features such as CMBs and markers of CAA. This approach allows clinicians to distinguish patients in whom oral anticoagulation may be resumed with caution from those in whom the risk of recurrent ICH outweighs the potential ischemic benefit.

LAAO emerges as a targeted strategy for patients at high hemorrhagic risk, particularly those with prior ICH, suspected or confirmed CAA, or recurrent bleeding in the setting of anticoagulation. This algorithm further accounts for procedural considerations, including whether patients are already undergoing cardiac surgery, thereby guiding the selection between pLAAO and surgical approach. By explicitly incorporating neurologic vulnerability, imaging phenotypes, and shared decision-making, this algorithm provides a practical roadmap for individualized care. Its application has the potential to define preventable hemorrhagic complications while preserving effective stroke prevention in a population for whom traditional anticoagulation strategies are frequently inadequate.

This decision-support algorithm presented in Figure 1 and Figure 2 represents a conceptual framework derived from the synthesis of the available evidence and expert interpretation rather than a validated clinical instrument. It has not been prospectively or retrospectively evaluated in independent clinical cohorts and should therefore be regarded as hypothesis-generating. The framework is intended to support multidisciplinary discussion and structured risk assessment in patients with competing thromboembolic and hemorrhagic risks. It is not meant to replace established clinical evidence, guideline-directed management, or individualized clinical judgment but rather to complement existing recommendations by incorporating neurologic vulnerability and imaging phenotypes into the decision-making process.

Ultimately, no randomized controlled trial specifically evaluating LAAO in patients with CAA, cerebral arteriopathy, or prior ICH has been completed. Current evidence in these high-risk populations is limited to observational studies and pooled analyses, which suggest LAAO may be feasible but requires cautious interpretation and randomized validation [106,118,119,120]. Ongoing trials, such as COMPARE LAAO and STROKE CLOSE, are enrolling patients with prior ICH or contraindications to anticoagulation but do not exclusively target CAA and have not yet reported results [106]. Therefore, management in these patients remains based on individualized risk–benefit assessment rather than definitive randomized evidence [106,118,119,120].

## 10. Conclusions and Future Directions

Stroke prevention in patients with AF and elevated ICH risk requires approaches that extend beyond traditional bleeding scores. Incorporating hemorrhage-specific factors and neuroimaging markers is essential to guide individualized management. While LAAO offers an important option for selected high-risk patients, its use should be grounded in multidisciplinary assessment rather than applied uniformly. This algorithm is intended as a decision-support framework to facilitate multidisciplinary discussion rather than to replace guideline-directed care. Future research should focus on validating ICH-specific risk tools, defining optimal post-procedural antithrombotic strategies, and evaluating outcomes across distinct hemorrhage phenotypes.

Finally, hemorrhage phenotyping fundamentally reshapes antithrombotic decision-making in patients with AF and prior ICH. Rather than treating prior ICH as a uniform contraindication, careful characterization of hemorrhage subtype, anatomic location, and neuroimaging markers allows clinicians to stratify recurrence risk with greater precision. Lobar ICH associated with CAA and high-risk MRI features may favor avoidance of long-term anticoagulation and consideration of LAAO, whereas deep hypertensive ICH with adequate blood pressure control may permit anticoagulation in selected patients. This biologically informed approach moves decision-making beyond traditional bleeding scores toward individualized, multidisciplinary care. For cardiovascular specialists, the core practical implications of hemorrhage phenotyping are summarized in Table 2 by clinical domain, key message, and clinical implications.

## Figures and Tables

**Table 1:** Perioperative risk, post-procedural OAC eligibility, and most common antithrombotic regimens following LAAO device implantation.

LAAO Device/Approach	Perioperative Risk	OAC-Free Eligibility After Procedure	Post-LAAO Antithrombotic Regimen
Non-percutaneous options			
LARIAT Hybrid Epicardial–Endocardial	- Pericardial effusion (2.2–10.4%) [91,92,93] - Cardiac perforation (2–2.7%) [93] - Major bleeding (4.5–9.7%) [92] - Pericarditis (2.4–2.7%) [93,94] - Need for urgent cardiac surgery (1.5–2.3%) [91,92,93] - Stroke (0.1–1.5%) [91,92,93,94]	No antiplatelet or oral anticoagulation in 19% [92] Range from 1.4–98% [93,95]	No consensus - 31% Aspirin monotherapy [92]; - 24% On dual antiplatelet therapy [92]; - 23% On oral anticoagulation [92]; - CHADS2 ≥ 2 remained on warfarin [94].
AtriClip	- Ischemic stroke: 0.45–1.7% [96,97] - Late TIA: 0.45–2.6% [96,97] - Hemorrhagic stroke 0.7–6.9% [97,98] - Device-related thrombus 7.2% [96]	43.7–100% [96] OAC at discharge: 65% [97] and OAC at F/U: 45.8% [97] 25% no OAC [98] - 35.9% on warfarin - 22.8% anti-aggregation - 12% on NOAC	Institutional consent [97]: - OAC stopped ≥3 months - After 2010: OAC stopped immediately after surgery
Surgical LAAO (Excision/Ligation during Cardiac Surgery)	Incidence of perioperative stroke was comparable between groups (2.1% vs. 2.6%) [99]. The risk of first ischemic stroke for LAAO cohort vs. no LAAO (4.6% vs. 6.9%, HR: 0.66, 95% CI: 0.52–0.84) [99] Similar rates of major bleeding, heart failure and perioperative death were similar amongst LAAO vs. no-LAOO groups [99].	- >80% on OAC at discharge in both groups [16] - >75% on OAC at 3 years follow-up [16]	Continued anticoagulation - 63.5% on vitamin K antagonist [83]; - 18.5% non-vitamin K OAC [83]; - 60.5% received OAC at all follow-up visits [83]
Percutaneous options			
AMPLATZER Amulet	- Pericardial effusion (2–4%) [100] - Cardiac tamponade (0.2–0.4%) [100] - Major vascular complications (1.3–2%) [100] - Device embolization (0.2–0.8%) [100] - Air embolism (19%) [100] - Device-related thrombus (1.6–3.3%) [13]	80% discharged with antiplatelet therapy alone [100] OAC at discharge 11.2% vs. 5.9% at 1 year vs. 6.6% at 2 years [100] At discharge [101]: - OAC: 8.2–12.4%; - DAPT: 80.4–85.2%. At 45 days [101]: - OAC: 9.2–10.7%; - DAPT 72.9–76.2%. - Single antiplatelet: 10.6–11.8%	(45 days–6 months): DAPT 45 days): DAPT [102] (>6 months): ASA indefinitely [102]
WATCHMAN FLX	- Pericardial effusion (0.42–1.23%) [103,104] - Major bleeding (1.08–2.05%) [103] - Procedure-related stroke (0.10–1.15%) [104] - Pericardial tamponade (0.29–4.3%) [92] - Device-related thrombus (0.4–7%) [103] - Device embolization (0.02–0.06%) [103] - WATCHMEN FLX significantly lower risk for major adverse events (HR: 0.84, *p* < 0.0001), ischemic stroke (HRS: 0.74, *p*: 0.02) and ischemic and systemic embolism (HR: 0.74, *p*: 0.0003) when compared to WATCHMEN 2.5 [105]	85–90% at 45 days [11]	(45 days–6 months): DAPT (>6 months): ASA indefinitely [102]; (3 months): DOAC ≥ 3 months: ASA indefinitely [102]; Does not tolerate short-term OAC? (6 months): DAPT ≥ 6 months: ASA indefinitely [102].

**Table 2:** Take-home messages for cardiovascular specialists.

Clinical Domain	Key Message	Practical Implication
Bleeding Risk Assessment	Not all ICH carries the same recurrence risk.	Prior ICH alone should not be treated as a uniform contraindication to anticoagulation.
Hemorrhage Phenotype	Lobar ICH, CAA, cSS, and multiple lobar microbleeds indicate markedly elevated recurrence risk.	In these patients, long-term anticoagulation may carry substantially increased hemorrhagic hazard, and alternatives such as LAAO should be considered.
Deep Hypertensive ICH	Deep ICH related to hypertensive arteriopathy has lower annual recurrence risk, particularly with strict blood pressure control.	Selected patients may be candidates for cautious anticoagulation resumption after multidisciplinary evaluation.
Neuroimaging Biomarkers	MRI markers (microbleeds distribution, siderosis, white matter disease) provide prognostic information beyond traditional bleeding scores.	Neuroimaging should function as a decision modifier in stroke prevention strategy selection.
Role of LAAO	LAAO should be considered based on hemorrhage subtype, imaging burden, and overall thromboembolic risk.	Decision-making should be multidisciplinary and individualized rather than score-based alone.

## Data Availability

The original contributions presented in this study are included in the article. Further inquiries can be directed to the corresponding author.
